# Comparison of pelvic C-clamp and pelvic binder for emergency stabilization and bleeding control in type-C pelvic ring fractures

**DOI:** 10.1038/s41598-021-81745-z

**Published:** 2021-01-27

**Authors:** Christof K. Audretsch, Daniel Mader, Christian Bahrs, Alexander Trulson, Andreas Höch, Steven C. Herath, Markus A. Küper, Working Group on Pelvic Fractures of the German Trauma Society

**Affiliations:** 1 Department for Traumatology and Reconstructive Surgery, BG Trauma Center, University of Tübingen, Schnarrenbergstraße 95, 72076 Tübingen, Germany; 2 Department of Trauma Surgery, BG Trauma Center, Murnau am Staffelsee, Germany; 3 Department of Orthopedics, Trauma and Plastic Surgery, University of Leipzig, Leipzig, Germany; 4 Department of Trauma, Hand and Reconstructive Surgery, Saarland University Hospital, Homburg, Germany; 5 Faculty of Medicine, Eberhard Karls University of Tübingen, Tübingen, Germany

**Keywords:** Health care, Medical research

## Abstract

Severe bleeding is the major cause of death in unstable pelvic ring fractures. Therefore, a quick and efficient emergency stabilization and bleeding control is inevitable. C-clamp and pelvic binder are efficient tools for temporary bleeding control, especially from the posterior pelvic ring. Yet the C-clamp requires more user knowledge, training and equipment. However, whether this makes up for a more efficient bleeding control, is still under debate. Patients with a type-C pelvic ring fracture were identified from the German Pelvic Registry (GPR) and divided into three groups of 40 patients (1. no emergency stabilization, 2. pelvic binder, 3. C-clamp). The matching occurred according to the parameters age, gender, initial RR and initial HB. Complication—and mortality rates were compared especially regarding bleeding control. Regarding ISS and fracture dislocation there was no difference. The use of the C-clamp resulted in more complications, a higher mortality rate due to severe bleeding and more blood transfusions were admitted. Moreover the pelvic binder was established noticeably faster. However, the C-clamp was more often rated as effective. There is no evidence of advantage comparing the C-clamp to the pelvic binder, regarding bleeding control in type-C pelvic ring fractures. In fact, using the pelvic binder even showed better results, as the time until established bleeding control was significantly shorter. Therefore, the pelvic binder should be the first choice. The C-clamp should remain a measure for selected cases only, if an adequate bleeding control cannot be achieved by the pelvic binder.

## Introduction

With approximately 3–8% of all fractures, pelvic fractures are rather rare^1^. The most important trauma mechanism for a pelvic ring fracture in young patients is a high energy trauma, while in older patients most pelvic ring fractures are caused by a low energy trauma^2^. However, because of the ageing society the incidence for pelvic ring fractures is constantly increasing^3,4^. Fractures of the posterior pelvic ring are of particular relevance, as large blood vessels, like the iliac arteries or the presacral venous plexus, are close to the fracture site. Therefore, severe bleeding problems can occur in type-C pelvic ring fractures. In about 80% of the cases these bleedings originate from a lesion of the presacral venous plexus or the fracture site itself^5^.

In the past years the overall mortality rate after pelvic fractures could be decreased to 5%^1^. However, among hemodynamically unstable patients the mortality rate can go up to 32%^6^. It has been proven, that unstable pelvic ring fractures with complete discontinuity of the posterior pelvic ring (type-C injuries according to the AO/OTA-classification respectively APCII-, APCIII- or VS-injuries according to Burgess/Young classification), have the highest probability of all pelvic fractures for potentially fatal bleeding^7–9^.

Treatment options of severely bleeding pelvic ring fractures range from non invasive procedures, like the pelvic binder, through minimally invasive procedures like the pelvic C-clamp or the external fixator or interventional radiological coiling, up to open surgical pelvic packing. In consequence, these treatment options vary drastically, not only regarding the invasiveness of the procedure, but also regarding the need for technical and infrastructural resources and the personal experience and training of the treating physician^10–12^.

Emergency stabilizations of the posterior pelvic ring, like the pelvic binder or the pelvic C-clamp, are used for both, fracture-reduction and, in consequence, to prevent further bleeding from the presacral venous plexus and the fracture itself. Moreover, they result in a stabilization of the intrapelvic volume and therefore the prevention of a potential fatal blood loss^10–12^. The pelvic binder has some advantages over the pelvic C-clamp: It is non-invasive, cheaper and applying the pelvic binder usually does not require specific training or equipment^10,12,13^. On the other hand, applying the pelvic C-clamp requires surgical skills as well as the experience to place the pins correctly; and after all the application is more time-consuming^14^.

These advantages of the pelvic binder might be the reason why its use has increased over the past years, whereas the pelvic C-clamp has been used less frequently^15^. In contrast, Schmal et al. showed that the invasive primary emergency stabilization of pelvic fractures with either a pelvic C-clamp or an external fixator is associated with a reduced mortality^15^ compared to the non-invasive primary care with the pelvic binder. In consequence, there has been a noticeable shift towards the pelvic C-clamp in injuries with increased severity as part of the damage control concept, as the pelvic C-clamp is considered the more effective therapy regarding bleeding control, especially in type-C pelvic ring fractures^10,15^.

Aim of this retrospective matched pair analysis from the German Pelvic Trauma Registry was to evaluate, whether the pelvic binder was equivalently suitable regarding bleeding control management as the pelvic C-clamp, especially in the case of type-C pelvic ring fractures.

## Patients and methods

We analyze data from the German Pelvic Trauma Registry of the DGU. Patient data of trauma patients who suffered from a pelvic fracture were collected prospectively in 30 German trauma centers between 2003 and 2017. The participating hospitals recorded anonymized data of treated pelvic fractures of this period, using standardized questionnaires. For this the patient's written informed consent was obtained from all subjects. The Ethics Committee of the Chamber of Physicians of the Federal State of Saarland approved the GPR (No. 29/14). Data acquisition and analysis was done following standards approved by the responsible ethics committee of the Eberhard-Karls-University in Tübingen, Germany [No. 351/2019BO2]. The study was retrospectively registered on 05/06/2020, (ClinicalTrials.gov, NCT04410952).

### Patient selection and matching

**Figure 1:**
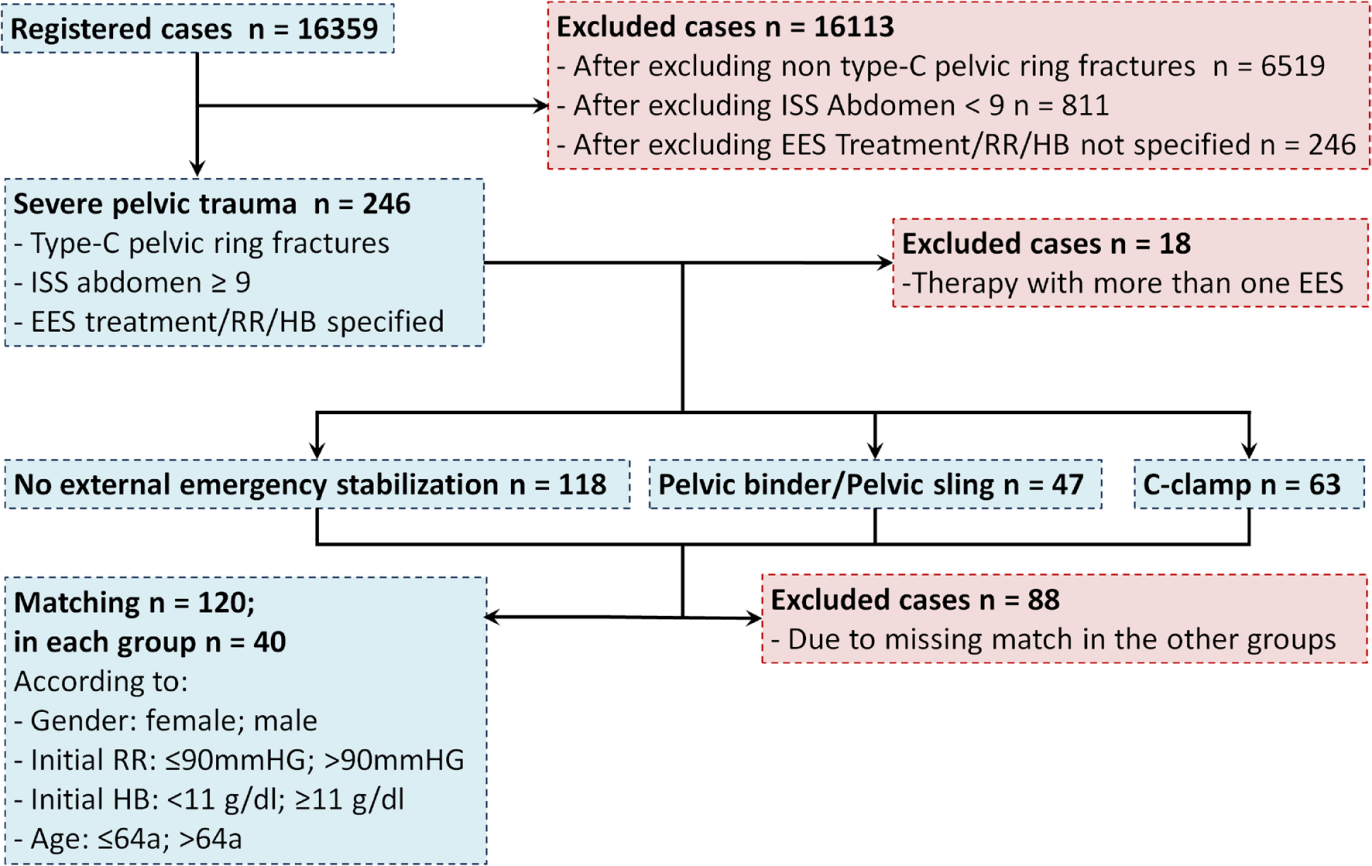
Patient selection, groups and matching. This diagram shows the included and excluded cases as well as the three compared groups and the parameters according to which the matching was carried out.

The total dataset includes 16,359 cases and was evaluated retrospectively. All patients with severe pelvic injuries, i.e. type-C pelvic ring fractures, were included. Then, patients with an ISS abdomen score of less than 9 were excluded, which left only patients in whom a potential intraabdominal bleeding must be considered. Cases in which the external emergency stabilization was not specified were left included. The remaining 246 cases were divided into the different treatment groups (Fig. 1): 1. Patients who received no external emergency stabilization. 2. Patients who were treated with a pelvic binder or a pelvic sling—the improvised equivalent with a sheet wrapped around the pelvis. 3. Patients who were treated with a C-Clamp. More than one therapy was documented in 18 cases, these data sets were excluded.

Age, gender, the initial HB and the initial blood pressure were used for matching the cases of the three groups. The age limit was set at ≥ 65 years. At this age, the functional requirements in everyday life usually decrease, whereas the comorbidities increase^16^. In polytraumatized patients an insignificant drop of the HB level can already indicate relevant bleeding. The German S3 polytrauma guidelines^17^,suggest to aim for a minimal HB level of at least 7–9 g/dl (4.4–5.6 mmol/l), thus in this study the HB threshold level was set at < 11 g/dl. The initial blood pressure threshold was set to 90 mmHg systolic RR, according to the shock definition in trauma patients^17^. At the end, 40 cases within each of the three groups were left for further analysis.

### Statistical evaluation

Differences in proportions of the categorical variables mortality, complications, additional treatments, and evaluation of the therapy success were evaluated with the Chi-squared—or the Fisher's exact test.

The distribution of the continuous variables fracture dislocation, total ISS and the transfusion requirement of packed red blood cells, but also the time until application of the external emergency stabilization, until definitive surgical treatment and until discharge from the hospital were assessed with Shapiro–Wilk test and turned out not to be normally distributed. Differences of these variables were therefore evaluated with the Wilcoxon signed-rank test.

The level of significance is set at 5% (p = 0.05). This is marked with an asterisk in the graphics. Two and three stars indicate a level of significance of 1% (p = 0.01) and 0.1% (p = 0.001) respectively.

All analyses were completed using RStudio, Version 1.2.5001^18^.

### Ethics approval

The Ethics Committee of the Chamber of Physicians of the Federal State of Saarland approved the GPR (No. 29/14). Data acquisition and analysis was done following standards approved by the responsible ethics committee of the Eberhard-Karls-University in Tübingen, Germany [No. 351/2019BO2]. The study was retrospectively registered on 05/06/2020, (ClinicalTrials.gov, NCT04410952).

### Consent to participate

The patient's declaration of consent was obtained respectively.

### Consent for publication (include appropriate statements)

The patient's declaration of consent was obtained respectively.

## Results

### Comparison of ISS and fracture dislocation

**Figure 2:**
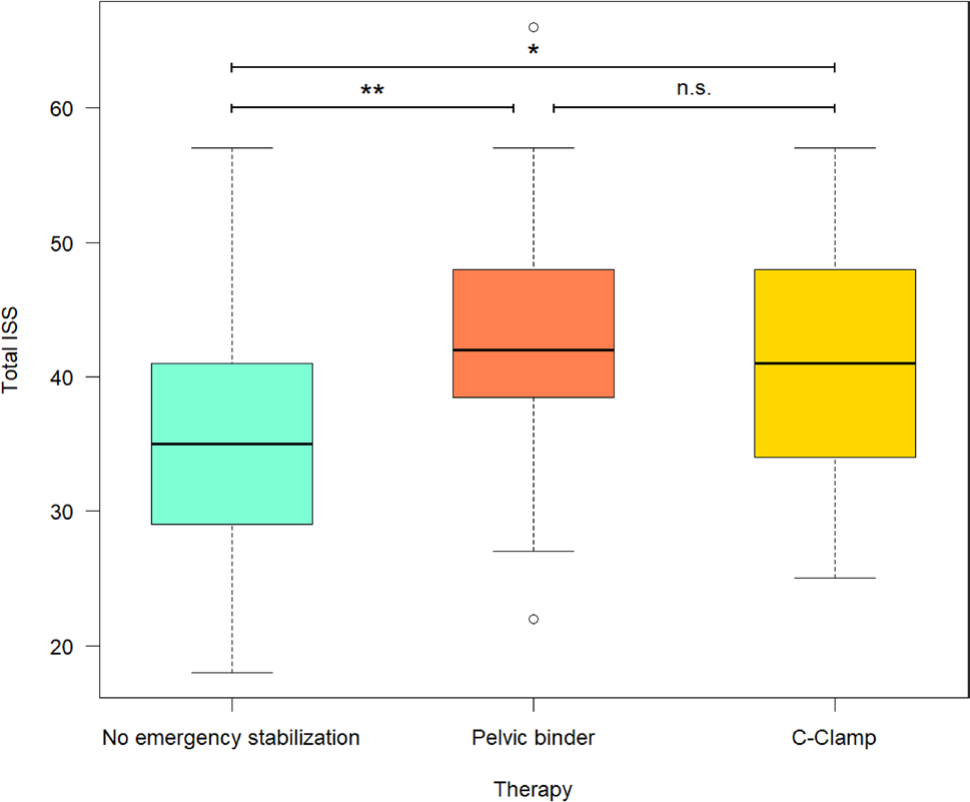
Comparison of total ISS. No significant difference (p = 0.188) can be found between the pelvic binder group (median ISS = 42) and the C-clamp group (median ISS = 41). In the group with no EES (median ISS = 35) a significant lower ISS can be found compared to the Pelvic binder group (p = 0.002) as well as to the C-Clamp group (p = 0.027).

There was no significant difference regarding the ISS (Fig. 2) and fracture dislocation between the C-clamp group (median ISS = 41) and the pelvic binder group (median ISS = 42). However, in the group without emergency stabilization (median ISS = 35) a significantly lower ISS could be identified compared to both, the pelvic binder group (p = 0.002) and the C-Clamp group (p = 0.027).

Regarding the fracture dislocation (for both, pelvic ring and sacrum), there was no significant difference between the pelvic binder group and the C-clamp group. However, in the group without any emergency stabilization procedure we found a significantly lesser fracture dislocation compared to the pelvic binder group but not the C-clamp group (pelvic ring: pelvic binder 27 mm, C-clamp 24 mm, no stabilization 17 mm p < 0.05 pelvic binder vs. no stabilization; sacrum: pelvic binder 9.5 mm, C-clamp 6 mm, no stabilization 5 mm, p < 0.05 pelvic binder vs. no stabilization.

### Time until emergency external stabilization and effectiveness of treatment

**Figure 3:**
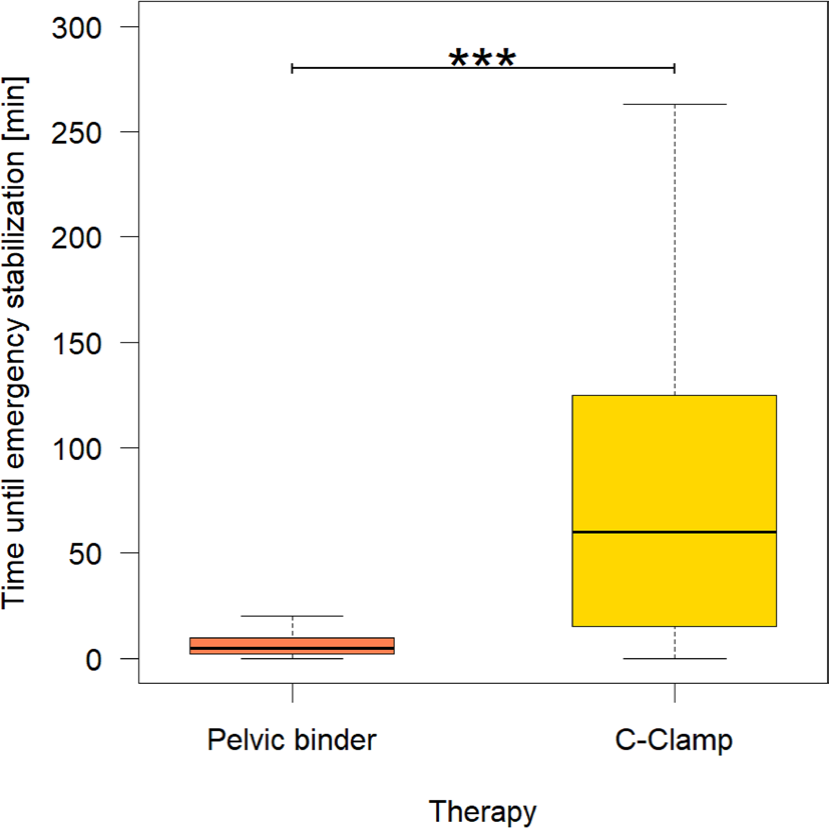
Minutes until emergency stabilization. The time until the emergency stabilization is applied is drastically and highly significant (p < 0.001) shorter for the pelvic binder (median 5 min) than for the c-clamp (median 60 min).

The median time until emergency external pelvic stabilization was significantly shorter in the pelvic binder group compared to the C-clamp group (5 vs. 60 min; p < 0.001). (Fig. 3).

**Figure 4:**
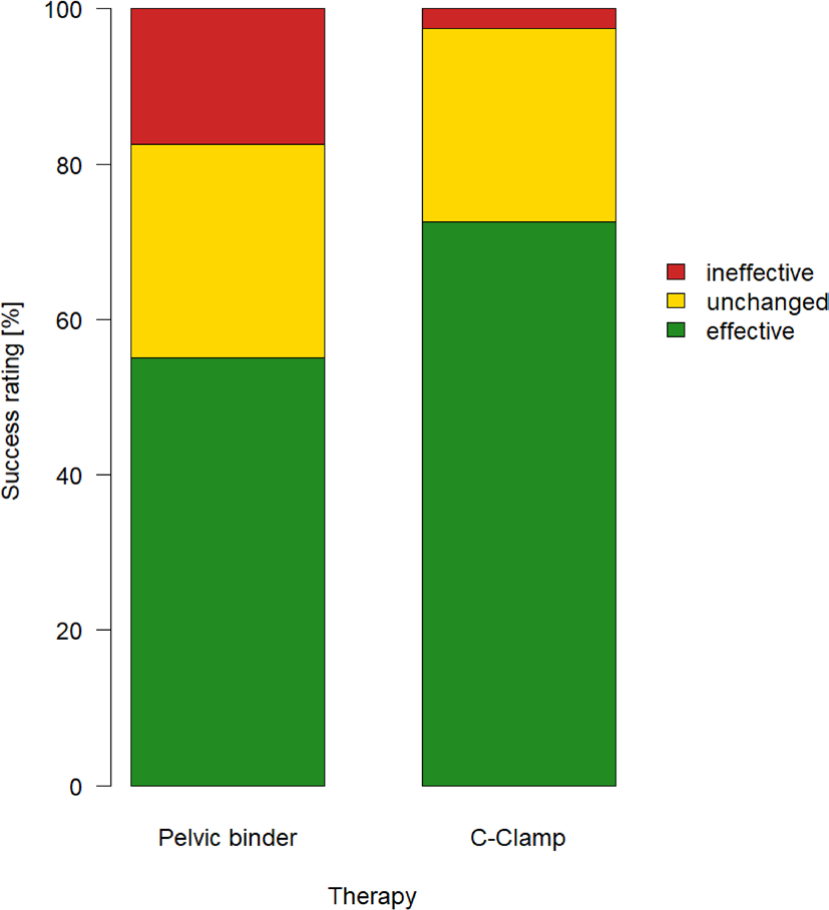
Rating of therapy success. Differences comparing the amount of evaluations as effective (pelvic binder n = 22; C-Clamp n = 29; p = 0.163), unchanged (pelvic binder n = 11; C-Clamp n = 10; p = 1) and ineffective (pelvic binder n = 7; C-Clamp n = 1; p = 0.057) turn out not to be significant.

**Figure 5:**
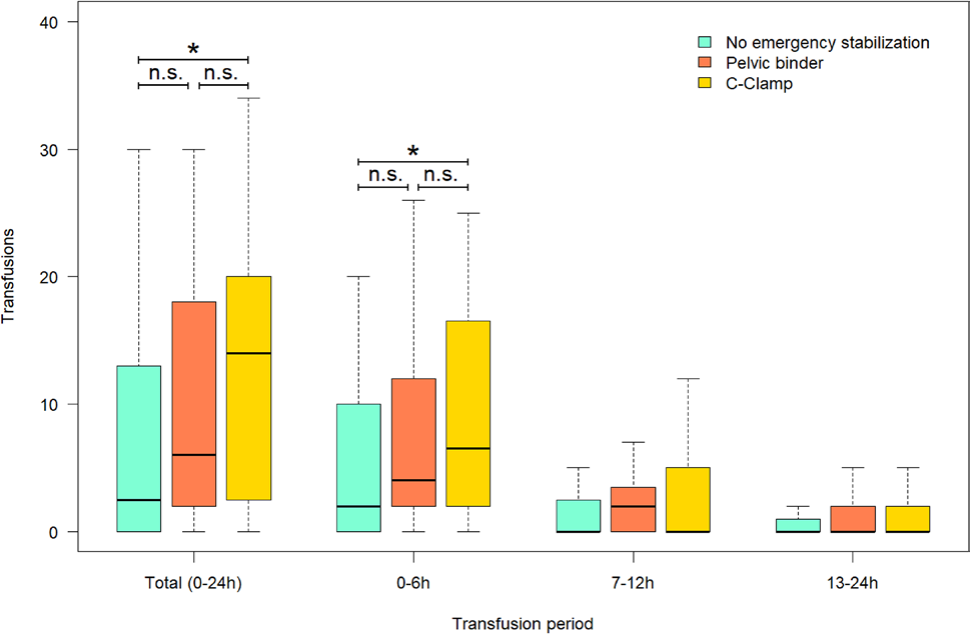
Need for transfusion of packed red blood cells. A significant difference persists in the comparison between the group without emergency stabilization (median 0–24 h = 2.5; 0–6 h = 2; 7–12 h = 0; 13–24 h = 0) and the group with c-clamp treatment (median 0–24 h = 14; 0–6 h = 6.5; 7–12 h = 0; 13–24 h = 0) only for the time periods 0–24 h and 0–6 h (0–24 h p = 0.015; 0–6 h p = 0.028; 7–12 h p = 0.109; 13–24 h p = 0.260). Not significant is the difference between the pelvic binder group (median 0–24 h = 6; 0–6 h = 4; XX 7–12 h = 2; 13–24 h = 0) and both, the C-clamp group (0–24 h p = 0.507; 0–6 h p = 0.143; 7–12 h p = 1; 13–24 h p = 0.276) and the group without emergency stabilization (0–24 h p = 0.138; 0–6 h p = 0.204; 7–12 h p = 0.256; 13–24 h p = 0.053). Outliers are not shown in this figure yet considered in the statistical calculations.

Though there was a tendency to subjectively rate the C-clamp more often effective than the pelvic binder, there was no statistical difference between the two groups (Fig. 4). Yet, looking at the number of transfused packed RBC, significantly more units of RBC were transfused in the pelvic C-clamp group than in the control group with no external emergency stabilization. In comparison to the pelvic binder group however, the difference was insignificant (Fig. 5). However, the higher number of transfused packed RBC was recorded only within the first 6 h after admission, there was no difference during the course of the following 18 h.

### Clinical course, bleeding complications and mortality

The overall respective complication rates were as high as 24% (16/40) in the group with no external emergency stabilization (EES), 52.5% (21/40) in the pelvic binder group and 65% (26/40) in the C-clamp group (not significant; n.s.). The mortality rates were as high as 10% (4/40) in the group with no EES, 20% (8/40) in the group with the pelvic binder and 22.5% (9/40) in the group with the C-clamp (n.s.).

Regarding bleeding complication rates, we found 2.5% (1/40) in the group with no EES, 7.5% (3/40) in the pelvic binder group and 17.5% in the C-clamp group (p = 0.05 no EES vs. C-clamp). The respective fatal bleeding complication rates were as high as 2.5% (1/40) in the group with no EES, 7.5% (3/40) in the pelvic binder group and 10% (4/40) in the C-clamp group (n.s.).

### Need for further emergency surgery of the pelvis

An external fixator was applied significantly more often in the pelvic binder group, compared to the C-clamp group (75% vs. 40%; p < 0.01). However, the need for an open emergency pelvic surgery was not different between the two groups.

### Clinical course

**Figure 6:**
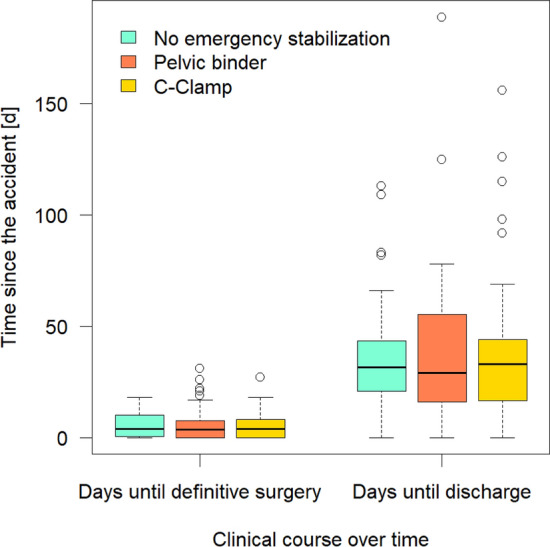
Clinical course over time with days until definitive surgery and discharge. There is no significant difference (no emergency stabilization vs. pelvic binder p = 0.831 and vs. C-clamp p = 0.617; pelvic binder vs. C-clamp p = 0.754) between the three groups in the time until definitive surgery (no emergency stabilization median = 4d, pelvic binder median = 3.5d, C-clamp median = 4d) When comparing the time until discharge (no emergency stabilization n = 31.5d, pelvic binder n = 29d, C-clamp n = 33d) there is no significant difference too (no emergency stabilization vs. pelvic binder p = 0.791 and vs. C-clamp p = 0.914; pelvic binder vs. C-clamp p = 0.856).

The median time until definitive pelvic stabilization was in the group with no EES 4 days, in the pelvic binder group 3.5 days and in the C-clamp group 4 days. The median length of Hospital Stay (LOS) was in the group with no EES 31.5 days, in the pelvic binder group 29 days and in the C-clamp group 33 days. Both, the time until definitive pelvic surgery and the LOS were not significantly different between the three groups (Fig. 6).

## Discussion

Fatal bleeding is the major cause of death in severe pelvic ring injuries. Whilst injuries to the anterior pelvic ring most often go along with bleedings from arterial damage (e.g. rupture of corona-mortis-vessel), bleedings due to posterior ring injuries occur in the vast majority of the cases from the fracture gap itself or from the presacral venous plexus^5^. External compression measures, like the non-invasive pelvic binder or the invasive pelvic C-clamp have proven to result in a stabilized intrapelvic space and therefore might reduce the potentially fatal loss of high blood volume^10–12^. However, whether the pelvic binder or the C-clamp provide better results in the management of bleeding control, especially in posterior pelvic ring injuries, still remains unclear.

Recently, Schmal et al.^15^ showed that mortality and complication rates after invasive emergency treatment (c-clamp and external fixator) are higher than in the non-invasive treatment group (pelvic binder). They attributed this observation to other risk factors, in particular age and ISS. However, in consequence they stated that the odds ratio, adjusted to these values, even shows a risk reduction after the invasive emergency treatment. In their study, both type-B and type-C pelvic ring injuries were included and external fixators and pelvic C-clamps were grouped together in the invasive treatment group. We evaluated the effectiveness and the complications of the C-clamp compared to the pelvic binder only in type-C pelvic ring injuries. Regarding the fracture dislocation, which is discussed to be an indicator of injury severity and bleeding risk^15,19^, we could not find a significant difference between the pelvic binder and the C-clamp. While the group matching ensured that age and gender, and especially the initial hemodynamic conditions, such as initial blood pressure and initial HB level, were not different between the two groups, injury severity as a possible confounder was ruled out, since only patients with an AIS ≥ 3 were included. Furthermore, the total ISS of the included patients showed no difference between the two groups.

We also found the tendency to subjectively judge the treatment in the C-clamp group more often effective than in the pelvic binder group. Previous studies already examined the subjective evaluation of the therapy success in detail and also over time, yielding comparable results^20^. However looking at objective parameters for bleeding control (e.g. numbers of packed red blood cells, time until their application, and fatal bleeding) we could not detect any differences, especially regarding fatal bleeding or the need for transfusions. On the contrary, there is a slight advantage of the pelvic binder over the c-clamp.

As shown previously and also in this study, bleeding represents a major cause of death after pelvic ring fractures^21^. This again underlines the relevance of the pelvic binder in the emergency treatment of pelvic fractures. Particularly the option of adequate bleeding control, compared to the C-clamp treatment, is of great advantage, and also reflected in our data, which might be especially due to the quicker application. This is due to the fact that the pelvic binder is simple to handle, which makes it also suitable for preclinical application. However, of course the correct application is important in the use of the pelvic binder, too. Incorrect positioning can significantly reduce its efficiency and can possibly also lead to complications^22^. Nevertheless, the use of the pelvic binder certainly remains easier than the application of the C-clamp.

Biomechanical studies of stability—and consequently the chance to achieve hemostasis in the sense of self-tamponading and clotting—, show equivalent results in the therapy with the pelvic binder and the C-clamp. However, both therapies are rated as inferior to any external fixator treatment^10,23^. According to our data, patients initially treated with the pelvic binder underwent a surgical application of an external fixator significantly more often, which may result in better rates of bleeding-related mortality and complications. Thus, the C-clamp might be neither the optimal solution in the context of emergency treatment, nor in the context of the subsequent mid- and long-term treatment. In the first case, the pelvic binder is surely superior due to its quicker and easier applicability and in the second case the external fixator is surely superior due to its higher stability. Yet, especially in type-C pelvic ring injuries, the C-clamp can play an important additional role together with the external fixator and, for example, pelvic packing as well as other treatment options, since it can be used to specifically address the posterior pelvic ring and is—in contrast to the pelvic binder—suitable for long-term use^10,24^.

The unexpected inferiority of the pelvic C-clamp with regards to mortality and complication rates, could be due to omission of therapies that are actually necessary, such as switching to the external fixator. These are performed more frequently after usage of the pelvic binder. This might be due to the fact that the pelvic binder is not allowed for long term use, but also due to the feeling of having performed a sufficient therapy after the more invasive C-clamp treatment. This is reflected in the finding, which is consistent with other studies, that C-clamp treatment is more often subjectively evaluated effective by the treating physicians^20^. Yet, according to our data, there is no evidence for a superior effectiveness of the C-clamp compared to the pelvic binder. This might explain why there is a numerical trend towards using the pelvic binder over the C-clamp. However, it remains to be noted that this trend is not always statistically significant due to the small sample size. Thus, further studies are desirable to address this issue. However, based on our data, the pelvic binder is equivalent to the pelvic C-clamp in terms of mortality and complications, especially those associated with bleeding in type-c pelvic ring injuries.

## Conclusion

In summary, there is no evidence for an advantage of the pelvic C-clamp over the pelvic binder, regarding bleeding control in type-C pelvic ring injuries. On the contrary, using the pelvic binder, by trend, shows better results than using the C-clamp. Moreover, the pelvic binder is easier and, especially, much quicker to use and apply. Therefore, to achieve immediate bleeding control in unstable pelvic ring injuries, rather the pelvic binder than the C-clamp should be used.

However, especially in the case of unstable type-C pelvic ring injuries, in which a timely definitive stabilization of the posterior pelvic ring is not possible due to other injuries (e.g. severe head injuries), the C-clamp might play an important role in the treatment concept, together with an external fixator stabilization of the pelvic ring. Thus, known complications related to the long-term use of a pelvic binder, like pressure marks or dislocation of the device, can be avoided, and ICU-care can be easily facilitated, for example because of a better accessible anogenital region. Primary use of the C-clamp should remain a treatment for selected cases only and if the trauma surgeon is familiar with it.
